# Conceptual approach to developing quality measures for transgender patients

**DOI:** 10.1186/s12913-021-06161-5

**Published:** 2021-02-16

**Authors:** Adam J. Rose, Michael S. Dunbar, Jaclyn M. W. Hughto, Guneet K. Jasuja

**Affiliations:** 1 Hebrew University School of Public Health, Jerusalem, Israel; 2 RAND Corporation, 4750 Fifth Avenue, Suite, Pittsburgh, PA 600 USA; 3 Brown University, Providence, RI USA; 4 Bedford VA Medical Center, Bedford, MA USA; 5 Boston University School of Public Health, Boston, MA USA; 6 OptumLabs Visiting Scholar, OptumLabs, Eden Prairie, MN USA

**Keywords:** Transgender persons, Quality of health care, Methods

## Abstract

**Background:**

Valid and reliable quality measures can help catalyze improvements in health care. The care of transgender patients is ripe for quality measurement, as there is increasing awareness of the increasing prevalence of this population and the urgency of improving the health care they receive. While best practices may not exist for some aspects of transgender health care, other aspects are characterized by well-developed and highly evidence-based recommendations. Our objective was to create a list of potential quality measures for transgender care.

**Methods and results:**

In consultation with our advisory panel, which consisted of clinical and academic experts in transgender medicine, we selected eight prominent clinical practice guidelines of transgender health care for review. Our four team investigators carefully reviewed all eight clinical practice guidelines. Through the course of multiple consensus-building meetings, we iteratively refined items until we had agreed upon a list of forty potential quality measures, all of which met the criteria for quality measures set forth in the Center for Medicare and Medicaid Services Blueprint for developing quality measures.

**Conclusions:**

This manuscript explains the origin of the quality measures we developed, and also provides a useful roadmap to any group hoping to develop quality measures for a field that has not previously had any.

## Background

### Conceptual basis

#### Why is it important to have quality measures?

The National Academy of Medicine (NAM) defines health care quality as “the degree to which health services for individuals and populations increase the likelihood of desired health outcomes and are consistent with current professional knowledge” [1]. Quality measures (QMs) are a diverse array of tools that are used to quantify performance on specific processes, outcomes, patient perspectives, and other factors associated with the provision and the goals of high-quality health care (i.e., safety, timeliness, effectiveness, efficiency, equity, and patient centeredness of care) [2, 3].

Since the 1980s, a steadily improving science and practice of quality measurement has driven remarkable improvements in the quality of care in a wide array of clinical domains [4]. However, there are areas of health care that have been relatively untouched by quality measurement [5], which may contribute to disparities or gaps in quality of care [6, 7]. The care of transgender patients is one of these areas. Implementing valid QMs can serve as a basis for improving care in a clinical area or population that previously lacked them [5, 8]. In light of the NAM call for research to advance the health of transgender patients [9]; we aimed to conduct research to identify candidate QMs for transgender care.

#### Why do we need quality measures for transgender care?

Transgender individuals, or people whose gender identity or expression differs from their assigned sex at birth, constitute a key population that would greatly benefit from quality measurement to improve the quality of their health care. Until recently, relatively little was known about the health and health care of transgender patients, in part because few data were systematically collected on this population. Existing research on the health and health care of transgender patients has largely relied on small convenience samples, heavily drawn from urban populations and specialty clinics dedicated to providing care for sexual and gender minority populations [10–14]. Despite these caveats, available data indicate disparities for transgender patients with respect to accessing and receiving high-quality health care [15, 16], and NAM and professional bodies such as the American College of Physicians have issued explicit calls to improve data collection and measurement, to drive improvements in the quality of health care delivered to transgender patients [17]. Although standards of care for transgender patients exist, to our knowledge, no previous effort has attempted to develop QMs for the care of transgender patients, or to use such QMs to examine the quality of care that is being delivered to such patients.

Although most health care provided to transgender patients is similar to that provided to cisgender (non- transgender) patients, transgender patients also have unique health care concerns. For example, some transgender patients undergo medical interventions (e.g., hormone therapy, gender-affirming surgeries) to bring their physical characteristics into better alignment with their gender identity – a process called medical gender affirmation. Best practices for delivering aspects of health care to transgender patients with high quality (i.e., “consistent with current professional knowledge”) are detailed in multiple clinical practice guidelines. As an example of a recommended aspect of care, which could be considered a potential quality of care measure, the Endocrine Society recommends that hormone levels should be checked within 3 months of starting hormone therapy, and then at least yearly thereafter [18, 19]; similar recommendations are found in many other guidelines [20, 21]. Another recommendation is that hormone levels should be kept in the physiologic range for the desired gender [18, 20, 22, 23]. Similarly, trans masculine (TM) spectrum patients (those who identify along the female-to-male spectrum), and trans feminine (TF) spectrum patients (those who identify along the male-to-female spectrum) who have taken hormone therapy, are at risk for osteoporotic fractures and should have bone mineral density measured at about age 65 [24]. However, other guidelines contain divergent views of which patients should have bone mineral density screening and at what ages [25]. These and similar aspects of transgender care are measurable, and improving the fidelity with which they are delivered may help improve population health for transgender patients. Our eventual aim is, therefore, to measure the quality of care received by a sample of transgender patients. Here, we describe the foundational work that may help to advance the process of developing QMs for this population.

#### How do we develop quality measures?

QM development is guided by a standardized approach, as set forth by the Centers for Medicare and Medicaid Services (CMS) Blueprint for Measure Development and Management [26]. The process begins with conceptual work, to form a list of candidate QMs that appear to meet at least some of these criteria. This conceptual work is the subject of the present manuscript.

According to the CMS Blueprint, the suitability of QMs is evaluated on four factors: 1) importance; 2) reliability and validity; 3) ease of understanding; and 4) feasibility of collection and calculation [26]. A QM that is important will ideally be based on strong scientific evidence that treating patients in this way will improve outcomes, although strong expert consensus may also be an acceptable basis for developing a QM [26]. A QM should be demonstrated to be reliable (has acceptably precise and consistent measurement) and valid (measures the intended aspect of care, not some other factor). A QM that is easy to understand will allow stakeholders (clinicians, patients, payers, and others) to use measure scores as a basis for improving care. Lastly, a QM that is feasible can be calculated based on easily available data, with a minimum of expense and effort. Sometimes there is a tradeoff among these four factors; for example, a measure of extreme importance in terms of potential to improve outcomes may be acceptable even if it requires considerable effort in terms of data collection. Indeed, some of the most important quality measurement efforts have been supported by chart reviews [27] – a process that is extremely effort-intensive but sometimes nevertheless necessary.

An example of a very successful quality measure, not specific to transgender care, which embodies all of these desirable attributes, is the proportion of patients admitted to the hospital with a coronary syndrome who received beta blockers while hospitalized. Widespread implementation of this measure in hospital settings led to such a marked improvement that it was eventually retired from use, as no further improvement was possible [4]. This success was driven, in part, because the measure embodied the four recommended characteristics. Specifically, the measure was important, in that beta blockers are clearly shown to improve outcomes for such patients [28] – a fact that is well-known and non-controversial. The measure was reliable and valid, in that its results truly gave hospitals an accurate barometer of whether they had improved in terms of consistently providing beta blockers to all such patients. The measure was easily understood and did not require complicated explanations or statistical knowledge to interpret its meaning. Finally, the measure was feasible to collect and to calculate, as hospitals keep track of all medications administered to patients and also of the admitting diagnosis. Thus, it was our aim to develop measures that would also embody these four qualities.

#### Guiding principles for quality measurement in transgender Health care

While the CMS Blueprint describes a general approach for developing and validating quality measures, we also had several guiding principles that are highly specific to our efforts to develop QMs for this specific population (i.e., transgender patients). These included:

1. Basing our prospective QMs on aspects of care whose clinical recommendations are ideally highly evidence based, or as a second-best, are characterized by a high degree of expert agreement combined with strong conceptual rationale.
2. Focusing on creating QMs that are specific to transgender patients (e.g., transgender hormone therapy to masculinize or feminize the body), rather than focusing on general care that would be provided to a patient of any gender (e.g., prevention and treatment of colon cancer).
3. Ensuring that the prospective QMs would apply to a sizable proportion of transgender patients, and not be so rare as to render the measure less useful.
4. Focusing on prospective QMs for which the data necessary to measure are likely to exist and be feasible. At this early stage, we did not exclude categories, but only ranked them in terms of likely or perceived feasibility of measurement; in later stages, we did eliminate some measures we collectively agreed would be extremely infeasible.

Our general approach to developing prospective QMs can be described as a “brainstorming” approach. We began with an extremely inclusive approach, aiming to include all possible QMs, while noting which ones seemed more or less feasible or promising. We then gradually narrowed down the number of measures through multiple stages of group discussion and consensus, as will be described below (Table 1). We had an expectation that even at the end of the process, we would still have many potential QMs, only some of which would eventually prove feasible. In other words, we began by trying to be as inclusive as possible and aimed to only slightly “filter” the measures prior to subjecting them to “testing” in an administrative dataset. In later stages of research, as will be described below, we would further narrow the number of QMs on the list based on the empirical results of such testing.

**Table 1:** Multistep process of developing a list of 40 potential quality measures for the care of transgender patients

No.	Step	Process/output
1.	Initial review of Clinical Practice Guidelines (CPGs)	Four reviewers, each made a complete list of all potential quality measures (QMs) contained in any of 8 CPGs
2.	Combining all four sheets into a single document, preserving all versions	One combined document, preserving all versions as recorded by four reviewers, allowing side-by-side comparisons of language: 120 potential QMs
3.	Combining versions into a single QM for each item	One combined document with a single proposed version of each potential QM
4.	Group discussions and refinement	One combined document with a single approved version of each potential QM – as agreed by all 4 reviewers by group consensus: 80 potential QMs
5.	Individual priority rankings	Each reviewer ranks all 80 potential QMs in terms of relative priority based on importance, feasibility of measurement, and other considerations
6.	Group priority rankings	Group discussion of priority rankings leads to a consensus-approved list of 40 potential QMs, ranked in order of priority
7.	Meetings with data analyst	Operationalization of all 40 potential QMs, with logic model, numerator and denominator, and codes to be used (diagnosis codes, medication lists, etc.)
8.	Convening of the Technical Expert Panel	Evaluation of our candidate QMs and recommendations of which of the measures might be best suited to serve as QMs

### Human subjects approval and informed consent

The present manuscript describes work that is not human subjects research; rather, it is essentially a review of the published literature and a consensus process among researchers. Therefore, human subjects approval and informed consent were not required.

## Methods and results

### Team and external expert advisors

Our team was co-led by a physician with expertise in quality measurement (AR) and an expert in hormone therapy and large database analyses (GJ). We were joined by an expert in transgender health and health care utilization (JH) and an expert in health psychology and quality measurement development and testing (MD). Our core team of four was advised by a panel of nine experts in transgender medicine, many of whom are active researchers and/or contributed to current clinical practice guidelines. We consulted with these experts via email and/or telephone at various important junctures during the process described below.

### Review of clinical practice guidelines

Care recommended as part of clinical practice guidelines (CPGs) may not always be suitable for direct adoption as quality measures, unless careful thought is given to how best to adapt them to this purpose [29]. However, CPGs remain a logical place to begin one’s search for which aspects of care are evidence-based and/or supported by a strong expert consensus [2, 26], and are integral to the initial information-gathering stage of new QM development. We accordingly began our search for potential QMs by reviewing what we considered to be the most important CPGs for the care of transgender patients. We recognize that these CPGs vary in terms of length, detail, approach to evidence-based medicine, clinical philosophy, and even perhaps perceived authoritativeness; indeed, a recent review has critiqued the rigor of these and other CPGs for transgender health care [30].

Our goal was to be as complete as possible in our search for potential QMs. That is, we aimed to identify all potentially important ideas at this stage, and to narrow them down later. Therefore, we reviewed eight particularly important CPGs, and consulted our advisory panel to ensure that we had not missed any CPGs at this stage. The eight CPGs that we reviewed were:

1. Callen-Lorde Community Health Center Protocols for the Provision of Hormone Therapy [31]
2. Endocrine Treatment of Gender-Dysphoric/Gender-Incongruent Persons: An Endocrine Society Clinical Practice Guideline [19]
3. Fenway Health - The Medical Care of Transgender Persons [32]
4. Sherbourne Health Centre Guidelines and Protocols for Hormone Therapy and Primary Health Care for Trans Clients [23]
5. UCSF Guidelines for the Primary and Gender-Affirming Care of Transgender and Gender Nonbinary People [20]
6. Endocrine Therapy for Transgender Adults in British Columbia: Suggested Guidelines [21]
7. Tom Waddell Health Center Protocols for Hormonal Reassignment of Gender [32]
8. The World Professional Association for Transgender Health Standards of Care for the Health of Transsexual, Transgender, and Gender Nonconforming People, 7th Edition [33]

Our initial approach to reviewing these CPGs was as follows (Table 1). All four members of our team read all of the CPGs in their entirety and identified each aspect of care that might have potential to serve as a CPG. At this point, we aimed for expansiveness and capturing all relevant ideas, leaving winnowing to future stages. Each of us kept a running flow sheet using Microsoft Excel (version 16.44, Microsoft Corporation, Redmond, WA), documenting in chronological order (i.e., from the start of each document through to the end) all relevant aspects of care. For each potential QM, we documented the following information:

- Category or subset of care
  - Hormone management: TF
  - Hormone management: TM
  - Cancer screening
  - Bone health
  - Cardiovascular health including venous thromboembolism
  - Physical examination and pelvic pain
  - Sexually transmitted infections including HIV
  - Preservation of fertility, fertility options
  - Sex reassignment surgery
  - Mental health care and consent before hormone therapy
  - Ways to be welcoming
  - Other (specify)
- Guideline (i.e., which of the 8 guidelines was the source)
- Page number
- Relevant text (cut and paste)
- Feasibility of measurement (4 levels, from very feasible to very infeasible, rated according to the subjective impression of the reviewer)
- Notes

At the end of this stage, each of us had compiled a lengthy spreadsheet containing all of the recommendations from the eight CPGs reviewed.

As a further stage of processing, we combined all four reviews into a single document, divided based on the twelve topics mentioned above. At the end of this step, we had created a list, for each topic, of all potential QMs. In many cases, we found the same potential QMs, but in other cases, fewer than all four of us had found a potential QM. Sometimes, we had identified the same QM, but had copied slightly different text about the potential QM. Creating a single unified document, while preserving all different versions of the text produced by all four reviewers, provided us with a substrate that we could later use to harmonize our disparate reviews of the same material.

#### Consensus-building and discussion

Having compiled this joint summary document, used it as a basis for combining or reconciling our disparate reviews into a single version of each potential QM. We divided the twelve topics among ourselves, and each of us took responsibility for three of them. Each of us removed or reconciled duplicate entries, aiming to keep (rather than discard) ideas that had been found by only some of us. At the end of this stage, we had a unified list of approximately 120 potential QMs.

Once we had done this, we held a series of weekly meetings. During these meetings, the one who was responsible for the topic area led the discussion and explained which version he or she had retained and how disparate versions had been harmonized into a single version. Group discussion sometimes led us to alter the text of the unified document. At this stage, we also collectively agreed to discard some ideas that were not suitable as potential QMs, because they were not measuring quality or because they were not even minimally feasible to be measured. We also eliminated some potential QMs that were not sufficiently specific to transgender care or that overlapped with existing quality measures aimed at the general population. At the end of this process, we were left with approximately 80 potential QMs, the text of which has been agreed to by all four investigators.

Our next step was to hold additional team discussions about the potential QMs. We further selected among them based on conceptual fit to serve as QMs, evidentiary basis, feasibility of measurement and availability of data, presence or absence of controversy about each aspect of care, and other related considerations. Without consulting with the others, we each ranked all the remaining potential QMs in terms of relative priority (based on feasibility of measurement, likely impact, and fit as a QM), and then compared our four lists. In the end, after discussion and consensus-building, our group agreed on a list of approximately 40 potential QMs, ranked in order of priority.

#### Brief summary of the 20 highest-ranked potential quality measures

As stated, at this point we had developed 40 potential quality measures that would be used as the basis for analyses. We present here the 20 highest-ranked measures in terms of priority, as ranked by our team’s internal discussions, as discussed in the previous section. The 20 potential quality measures are briefly summarized in Table 2. It is important to note that not all of these potential quality measures were ultimately successful. Some of them could not be operationalized due to a lack of suitable data. For example, we were unable to confidently identify cyclical preparations of estrogen in the data (Measure 2), and this measure was abandoned. All measures were critiqued by the technical expert panel (TEP) later when we presented the results of our initial analyses, with an eye toward improving future versions of them. This is the topic of a separate, forthcoming manuscript.

**Table 2:** Brief summary of 20 highest-ranked potential quality measures developed by our team based on review of eight prominent clinical practice guidelines

	Title	Category	Denominator	Numerator
Measure 1.	Receiving ethinyl estradiol formulations (not recommended)	Hormone Management (TF).	TF patients who received an estrogen formulation	Patients who specifically received an ethinyl estradiol formulation
Measure 2.	Receiving cyclical preparations of estrogen therapy (not recommended)	Hormone Management (TF).	TF patients who received an estrogen formulation	Patients who received cyclical preparations
Measure 3.	Measurement of testosterone and estradiol levels after starting estrogen therapy	Hormone Management (TF).	TF patients who received an estrogen formulation for feminizing therapy	Patients who had a testosterone level and/or an estradiol level measured within 3 months of starting therapy, and twice within 6 months
Measure 4.	Measurement of testosterone levels after starting testosterone therapy	Hormone Management (TM).	TM patients who received a testosterone formulation for masculinizing therapy	Patients who had a testosterone level measured within 3 months of starting therapy, and twice within 6 months
Measure 5.	Supratherapeutic testosterone levels (not recommended)	Hormone Management (TM).	TM patients who received a testosterone formulation for masculinizing therapy	Patients who recorded a testosterone level above the normal male physiologic range
Measure 6.	Supratherapeutic estradiol levels (not recommended)	Hormone Management (TF).	TF patients who received an estrogen formulation for feminizing therapy	Patients who recorded an estrogen level above the normal female physiologic range
Measure 7.	Measurement of metabolic panel after starting spironolactone.	Hormone Management (TF).	TF patients who received spironolactone for feminizing therapy	Patients who had measurement of K, Cr, and BUN within 3 months of starting spironolactone
Measure 8.	Measurement of metabolic panel after increasing the dose of spironolactone	Hormone Management (TF).	TF patients who received spironolactone for feminizing therapy and experienced a dose increase	Patients who had measurement of K, Cr, and BUN within 3 months of increasing the dose of spironolactone
Measure 9.	Measurement of Hb and HCT after starting testosterone	Hormone Management (TM).	TM patients who started testosterone for masculinizing therapy	Patients who had measurement of Hb and/or HCT within 3 months of starting testosterone therapy
Measure 10.	Patients with a history of hyperkalemia or renal insufficiency should not be given spironolactone	Hormone Management (TF).	TF patients who began spironolactone for feminizing therapy	Patients who had a history of hyperkalemia or renal insufficiency prior to beginning spironolactone
Measure 11.	Adequate laboratory testing prior to beginning spironolactone	Hormone Management (TF).	TF patients who began spironolactone for feminizing therapy	Patients who had undergone measurement of potassium, BUN, and/or creatinine during the prior 6 months
Measure 12.	Adequate laboratory testing prior to beginning testosterone	Hormone Management (TM).	TM patients who began testosterone for masculinizing therapy	Patients who had undergone measurement of Hb and/or HCT during the prior 6 months
Measure 13.	Hormone therapy should not be given to patients with a history of hormone-responsive cancer	Hormone Management (TF and TM).	Patients who began estrogen or testosterone for feminizing or masculinizing therapy	Patients who had a history of breast, prostate, ovarian, or testicular cancer prior to beginning therapy
Measure 14.	Pap smear not appropriate below age 21	Cancer screening	TM patients age 18–20	All such patients who received a Pap smear prior to age 21
Measure 15.	Bone densitometry needed for trans patients	Bone health	For TF: those who received at least 180 days of feminizing therapy For TM: all	DEXA scan at least once between age 60–70
Measure 16.	Estrogen should be given by the transdermal route in patients with elevated cardiovascular risk	Cardiovascular health	Recipients of estrogen for feminizing therapy, divided into cardiovascular risk categories based on comorbidities	Estrogen by transdermal vs. oral route
Measure 17.	Estrogen should be given by the transdermal route in patients with prior venous thromboembolism	Cardiovascular health	Recipients of estrogen for feminizing therapy with history of prior venous thromboembolism	Estrogen by transdermal vs. oral route
Measure 18.	Oral testosterone preparations are no longer recommended	Hormone Management (TM).	Recipients of testosterone for masculinizing therapy	Patients who received testosterone via the oral route (rather than transdermal or intramuscular)
Measure 19.	Spironolactone should not be given, or should be given with caution, in those taking an angiotensin-converting-enzyme (ACE) inhibitor or angiotensin receptor blocker (ARB)	Hormone Management (TF).	Patients who received spironolactone for feminizing therapy	Patients who also received an ACE inhibitor or ARB concurrently
Measure 20.	Mammography should be performed every 24 months for TM patients and for TF patients who received estrogen for feminizing therapy	Cancer screening	All TM patients, and those TF patients who received estrogen for feminizing therapy for at least 180 total days	Screening mammography at least every 24 months

For the purposes of illustration, we will briefly discuss a representative measure - Measure 3. Measure 3 applies to patients who received feminizing therapy with an estrogen preparation. The measure states that they should have estradiol and testosterone levels measured once within 3 months of starting therapy, and twice within 6 months of starting therapy. This measure was based in part on a recommendation from the UCSF Guideline [20], but similar recommendations were found in several other CPGs [19, 21, 32]. The CPGs differed with respect to the suggested frequency and timing of such monitoring as well as recommendations to check levels if the patient appears to be responding to therapy as expected. These disagreements among guidelines were later echoed as discussion points among members of the TEP during the group discussion.

The denominator (people eligible for the measure) consisted of all people who had received feminizing therapy with estrogen (spironolactone therapy alone would not require monitoring of testosterone and estradiol levels). We intentionally focused on people who had a 6-month period without estrogen preceding their first prescription (a medication-free period), to ensure that we would be measuring new prescriptions. We also required patients to receive at least 6 continuous months of therapy to be part of this measure. For the numerator, we then tabulated which patients had a testosterone level and/or an estradiol level measured, at least once within 3 months of the start date for estrogen therapy, and at least twice within 6 months of the start date. Similar discussions for the other measures can be found in Table 2.

#### Brief overview of the data used for operationalization of potential quality measures

Here, we briefly discuss the data we used to operationalize the potential quality measures we developed and our previous work with this data to identify our transgender cohort. We used administrative data from the OptumLabs® Data Warehouse (OLDW), which includes de-identified claims data for commercially insured and Medicare Advantage enrollees. OLDW includes data on approximately 160 million unique individuals. The detailed patient-level information comprises more than 2500 data elements, including enrollment, medical claims, pharmacy claims, and lab results across a variety of care settings. Medical claims include both International Classification of Disease (ICD) codes and Current Procedural Terminology (CPT) codes. The claims data are supplemented by public, private, and self-reported health and sociodemographic information. Our study sample included 74 million adults enrolled in commercial or Medicare Advantage plans between 2006 and 2017 who were ≥ 18 years and had a claim initiated during this study period.

Our group had already performed considerable work with this database to identify transgender patients within it, and to further categorize them as TM, TF, or as unknown (transgender but not clearly TM or TF). Our approach to this is described in detail in a separate publication [34]. Briefly, using a mixture of transgender-related diagnosis and procedure codes, and use of hormone therapy, we identified 27,227 unique transgender people in the database who would form the basis for our quality measurement efforts to follow. Of these 27,227 transgender people, 8694 (32%) were identified as trans masculine (TM), 3959 (15%) were trans feminine (TF), and 14,574 (54%) could not be classified (unknown) [34].

While interested readers are encouraged to refer to the other publication for details, we will give brief examples here of how we might have identified some representative patients as transgender. A patient might be identified as TM based on having a diagnosis code for gender identity disorder, female gender marker, and receiving high-dose testosterone therapy for a prolonged period of time (higher dose than would be used for off-label treatment of hypoactive sexual desire). Similarly, a patient might be identified as TF based on having a diagnosis code for gender identity disorder, having a male gender marker, and having undergone a surgery suggestive of TF status, and receiving estrogen and high-dose spironolactone [34].

#### Operationalization of potential quality measures

In order to prepare for the identification of potential QMs in the OLDW, our group held a series of meetings with a data analyst to jointly work on how to operationalize each of the potential QMs as an actual analysis, with an intention of eventually preforming such analyses among the group of 27,227 transgender patients that we had identified. During these meetings, we frequently referred to the text of the clinical practice guidelines that we had used as the source for our QMs, to ensure that our measures would closely conform to the wording of the guidelines. Operationalizing measures involved defining the denominator and the numerator for each QM. During this step, we were sensitive to the limitations of large databases in general and the specific details of the database that we would be using. We iteratively explored which information would be needed to analyze each measure, such as lists of ICD codes, CPT codes, medication codes, laboratory codes, or other data.

Between meetings, members of our team worked on compiling lists of such codes, based on published literature when it was (rarely) available, or based on original compilation when it was not. For example, we compiled lists of ICD codes for major depression, for cardiac risk factors, and for hyperkalemia, and lists of CPT codes for screening mammography and mastectomy. Our team and the data analyst worked together, over the course of weeks, to fill in these gaps, until we were satisfied that we had a complete set of codes and a complete logic model for defining each of the 40 proposed analyses. This included having a plan for exceptions (i.e., patients who would be exempt from the measure) and a plan to address perceived challenges of data quality. We also defined which measures would apply specifically to TF patients, which would apply specifically to TM patients, and which could apply to any transgender patient.

#### Plans for initial testing and technical expert panel

While these next steps are outside the scope of the present manuscript, we provide a brief summary of them below to contextualize how the efforts described above fit into our long-term goal of developing quality measures for transgender care. Over the succeeding months, we completed analyses for each of the 40 proposed QMs, including the denominator (how many patients were eligible for the measure), the numerator (how many had fulfilled the measure), and other relevant details such as how the measure varied over time or by geographic region. We then created a brief summary of the results and a discussion guide and used this as the basis for convening a technical expert panel (TEP). The TEP was charged with evaluating our results and, based on the results and their outside knowledge, recommending which of the measures might be best suited to serve as QMs. These recommendations, and the TEP discussion, were organized around the four dimensions of a quality measure from the CMS Blueprint [26], as discussed above. The results of our analyses, and the proceedings of the TEP, will be published in future manuscripts.

## Discussion

This paper described the process that our group used to identify potential QMs for the care of transgender patients. While the process of QM development was not unique to our project, our intent is to provide a reference for others to understand the work we did in its broader context and to potentially help others to plan similar efforts to identify QMs for other fields of study. Other aspects of our process, such as the need to develop methods for identifying transgender individuals in automated administrative datasets, were unique to this clinical area and the context of the field of transgender medicine at the present time. We suspect this would be true of other efforts as well – there are commonalities for how any team would go about developing QMs, while there remains a need to recognize what is unique about each field of study and to incorporate such an understanding into the effort.

One noteworthy limitation of this effort is that most of the measures we identified may not apply to gender non-binary patients. In part, this may be because the CPGs that we reviewed have a fairly non-prescriptive approach to the care of such patients, or it may be that conventional data sources are not sufficient to characterize the care of nonbinary people. In addition, our measures focus much more on the care of patients who desire hormone therapy and not all transgender people utilize hormones to affirm their gender. Again, our measures focused on measuring the sorts of care for which we have data. Future efforts should focus on developing quality measures for these other populations as well, to the extent that it would be possible to do so.

Another limitation is that, while we reviewed eight particularly important CPGs for the care of transgender patients, there are also others that we did not review [35–38]. At the beginning of our project we consulted with nine experts in the field of transgender medicine about choosing these CPGs, and at the time, they did not recommend any sources beyond these. While some of the content in the CPGs that we did not review overlaps with the content in the CPGs that we did review, there also may have been non-overlapping material that could have led to additional potential quality measures, beyond those we developed.

## Conclusions

It is important to understand that the process described in this paper, and even the forthcoming results of our analyses and the TEP proceedings, are more akin to the “end of the beginning” than to the “beginning of the end” of the process of developing QMs for transgender care. Even those QMs that are recommended by the TEP for further development will still require extensive testing and refinement before they are ready for real-world use in actual care settings [26]. Nevertheless, every journey must begin with a first step, and we are proud to have contributed a first step to this one. It is our hope that these measures, when fully refined and eventually deployed, will contribute to improving the quality of care delivered to transgender patients.

## Data Availability

The authors will send any of the materials used in creating the candidate quality measures upon request.

## Abbreviations

CMS: US Centers for Medicare and Medicaid Services
CPGs: Clinical Practice Guidelines
CPT: Current Procedural Terminology Codes
ICD: International Classification of Diseases Codes
NAM: National Academy of Medicine
OLDW: OptumLabs Data Warehouse
QM: Quality Measure
TEP: Technical Expert Panel
TF: Transfeminine
TM: Transmasculine
